# Concurrent Radiotherapy with Carboplatin and Cetuximab for the Treatment of Medically Compromised Patients with Locoregionally Advanced Head and Neck Squamous Cell Carcinoma

**DOI:** 10.3389/fonc.2014.00165

**Published:** 2014-06-30

**Authors:** Kunal Saigal, Edgardo S. Santos, Khaled Tolba, Deukwoo Kwon, Nagy Elsayyad, Matthew C. Abramowitz, Amar Mandalia, Michael Andrew Samuels

**Affiliations:** ^1^Department of Radiation Oncology, University of Miami School of Medicine, Miami, FL, USA; ^2^Division of Medical Oncology, Lynn Cancer Institute, Boca Raton, FL, USA; ^3^Division of Medical Oncology, University of Miami School of Medicine, Miami, FL, USA; ^4^Department of Epidemiology and Biostatistics, University of Miami School of Medicine, Miami, FL, USA; ^5^University of Miami School of Medicine, Miami, FL, USA

**Keywords:** carboplatin, cetuximab, radiation, head, neck, squamous cell carcinoma

## Abstract

**Background:** Cetuximab (Cx) + radiation therapy (RT) is well-tolerated and has improved survival in patients (pts) with locoregionally advanced head and neck squamous cell carcinomas (LA-HNSCC). However, its efficacy when compared to HD-DDP + RT has been questioned. At our institution, low-dose weekly carboplatin is added to Cx + RT for patients unsuitable for HD-DDP.

**Methods:** We reviewed records of 16 patients with LA-HNSCC treated with definitive Cx + carboplatin + RT at the University of Miami from 2007 to 2011. Median follow-up was 24 months (range: 1–69 months).

**Results:** Median age: 71.5 years (range: 57–90 years); 15 male, 1 female. ECOG PS 0 = 15, 1 = 1. TNM staging was: *T*_1_ = 1, *T*_2_ = 5, *T*_3_ = 8, *T*_4_ = 2; *N* stage: *N*_0_ = 8, *N*_1_ = 5, *N*_2a_ = 2, *N*_2b_ = 1. All patients received weekly carboplatin (AUC 1.5–2), Cx given conventionally and daily conventionally fractionated RT. Median total weeks of concurrent systemic therapy = 7 (range: 3–8 weeks). RT was delivered to a median total dose of 70 Gy (range 30–74 Gy). Of the 15 evaluable patients, there were: 12 CR, 2 PR, and 1 PD. There were three local in-field failures, two regional failures, and three distant failures. At last follow-up, 8/15 patients remained with NED. Three-year locoregional recurrence was 28.3% (95% CI: 7.7–53.9%). Mean percentage of weight loss was 14% (range: 6–26%). Two patients required systemic therapy dose reduction. Three patients experienced a treatment delay and three did not finish RT as planned including a patient who received only 30 Gy due to death secondary to MI during treatment.

**Conclusion:** In this small retrospective series, carboplatin/Cx/RT was well-tolerated and efficacious in patients unsuitable for HD-DDP having LA-HNSCC. Acute toxicities were similar to Cx + RT, likely due to the non-overlapping toxicity profiles of the two systemic agents. We hypothesize that the addition of a well-tolerated cytotoxic chemotherapy agent may improve the therapeutic ratio of Cx + RT in patients who are poor candidates for more aggressive therapies and warrants evaluation in a prospective manner.

## Introduction

In the past two decades, there have been important advances in the management of locoregionally advanced head and neck squamous cell carcinomas (LA-HNSCC). The efficacy of radiation therapy (RT) with concurrent chemotherapy has altered the treatment approach of these patients from aggressive surgery to one that allows for organ preservation, thereby decreasing cosmetic and functional impairments while maintaining comparable long-term control rates to strategies involving definitive surgical resection. Based upon the results of several phase III randomized controlled trials as well as a large meta-analysis, concurrent chemoradiation therapy has become the standard of care approach for patients seeking non-surgical therapy (1–6). For this group of patients, a radiation dose to gross disease of approximately 70 Gy is commonly used with concurrent high-dose cisplatin-based chemotherapy (CRT). While overall survival (OS) is superior with such regimens compared to treatment with radiation alone, the addition of chemotherapy adds to the mucosal, gastrointestinal, and metabolic toxicity of treatment.

Unfortunately, a sizeable proportion of LA-HNSCC patients, particularly those who are elderly, those with compromised performance status, or those with significant medical comorbidity, may not be appropriate candidates for full-dose concurrent cisplatin-based chemoradiation therapy. For these patients, alternative choices of less toxic systemic therapy in combination with RT are attractive therapeutic options. The epidermal growth factor receptor (EGFR, ErbB-1) has been found to be expressed in virtually all head and neck squamous cell carcinomas. Binding of growth factor ligands to EGFR leads to its constitutive activation, which results in changes in gene expression, anti-apoptotic activity, and increased cell proliferation (7). Cetuximab (Cx) (Ertibux^®^, Bristol–Myers Squibb) is a chimeric monoclonal antibody that binds to the ligand-binding domain of EGFR and inhibits receptor activation, thereby preventing the aforementioned events that lead to tumor progression. It is also postulated to have radiosensitizing effects (8).

Results from a randomized phase III trial by Bonner et al. have shown that the addition of Cx to RT alone leads to improvements in all clinically relevant outcomes, including OS (9). However, combination cetuximab/RT has not been compared to CRT using conventional cytotoxic agents in a randomized setting, and recent retrospective data suggest inferior outcomes with Cx/RT when compared with CRT (10, 11). Despite this, Cx/RT remains the treatment regimen of choice at many institutions for patients not suitable for CRT. At our institution, the perceived underwhelming results with Cx/RT have led us to add an additional low-dose systemic radiosensitizing chemotherapy agent with a non-overlapping side effect profile (carboplatin) to Cx/RT on a case-by-case basis for patients who are not suitable to receive CRT in an effort to improve outcomes without adding excessive toxicity. Carboplatin was chosen because it has been utilized by some investigators as the backbone of concurrent systemic therapy with RT in multiple published studies (12, 13). We now report the toxicity and clinical efficacy of combination carboplatin/cetuximab/RT (CCRT) and provide the first report in the literature using this regimen, in order to determine if it is suitable for further prospective evaluation.

## Materials and Methods

This retrospective review was approved by the University of Miami Institutional Review Board. We reviewed the medical records of patients treated with concurrent CCRT at the University of Miami Sylvester Comprehensive Cancer Center between 1/2007 and 12/2011. In total, 16 patients were identified who presented with newly diagnosed, biopsy-proven non-metastatic squamous cell carcinomas of the oropharynx, larynx, or hypopharynx who then completed a course of organ-preserving therapy with this regimen. Patients were offered this treatment regimen primarily due to advanced age and/or frailty, baseline abnormalities in organ function such as chronic renal insufficiency or hearing loss that would preclude the safe use of cisplatin, or poor functional reserve and/or social support that would render completing a course of CRT excessively difficult.

All patients were assigned a performance status using the ECOG criteria (14). Staging was performed clinically as per the AJCC 2010 guidelines (version 7.0) (15). Toxicities were graded using the National Cancer Institute Common Terminology Criteria for Adverse Events v4.0 (NCI-CTCAE 4.0) (16). Patients were encouraged, but were not required, to undergo prophylactic feeding tube placement.

Systemic therapy was delivered using a loading dose of Cx at 400 mg/m^2^ 1 week prior to initiating RT. At the start of RT and thereafter, Cx was given weekly at 250 mg/m^2^ along with weekly carboplatin at an AUC of 1.5–2 mg/ml/min. RT was delivered using megavoltage linear accelerators to deliver conventionally fractionated RT at 2 Gy/fx to a total dose of approximately 70 Gy to gross disease in 33–35 fractions delivered once-daily on weekdays. Generally, patients were treated using intensity modulated radiation therapy (IMRT) using dose-painting techniques. Following therapy, patients were observed with routine clinical examination combined with radiographic imaging for disease surveillance. Imaging typically included a contrasted neck CT scan at 6 weeks from the completion of CRT and a PET–CT scan at 12 weeks from the completion of CRT. For persistent disease and/or disease progression, surgical salvage was offered if patients were deemed to be appropriate candidates. Study endpoints related primarily to toxicity and efficacy. Specific toxicity endpoints were Grade 3–5 mucositis, rash, dermatitis, dysphagia, and xerostomia.

The follow-up duration was calculated starting on the date of diagnosis. The date of progression was selected as the date of first event including locoregional recurrence (LRR), distant metastasis, or death. LRR was defined as recurrence at the primary site or in the neck after a disease-free interval. Patients who were alive without evidence of progression were censored at the date of last contact. OS was defined as the time from diagnosis to death from any cause with surviving patients censored at date of last contact. Progression-free survival (PFS) and OS were estimated by the Kaplan–Meier method.

## Results

### Patient and tumor characteristics

Patient and tumor characteristics of the cohort are summarized in Table 1. We identified a total of 16 patients who were treated with a course of definitive concurrent CCRT. Median age at diagnosis was 71.5 years (range: 57–70 years); 15/16 were male. At baseline, all patients had an ECOG performance status of 0 or 1. The race/ethnicity of the patients studied was as follows: 3 White/Non-Hispanic, 11 White/Hispanic, and 2 Black. Medical comorbidities were common in the cohort and their incidence is summarized in Table 2. All patients selected to receive this regimen had at least one chronic medical condition. The medical oncologists involved assessed the candidates globally in terms of aggregate medical comorbidities and did not restrict the regimen specifically to patients with a decreased baseline performance status, but rather to patients felt to be frail and therefore have inadequate functional reserve to withstand more aggressive therapy. A gastrostomy tube was placed in 9/16 patients, with no patient requiring any other form of enteral nutrition.

**Table 1:** **Patient and disease characteristics**.

Variable	*N*	%
Total patients	16	
**AGE AT DIAGNOSIS**		
Median (min, max)	71.5 (57, 90)	
**SEX**		
Male	15	94
Female	1	1
**RACE/ETHNICITY**		
White/non-Hispanic	3	19
White/Hispanic	11	69
Black	2	12
**DISEASE SITE**		
Tonsil	4	25
Base of tongue	5	31
Supraglottic larynx	2	12
Larynx	4	25
Hypopharynx	1	6
***T*** ***STAGE***		
*T*_1_	1	6
*T*_2_	5	31
*T*_3_	8	50
*T*_4_	1	6
*T*_x_	1	6
***N*** ***STAGE***		
*N*_0_	8	50
*N*_1_	5	31
*N*_2a_	2	12
*N*_2b_	1	6
*N*_2c_	0	0
*N*_3_	0	0
**GROUP STAGE**		
I	0	0
II	1	6
III	10	63
IVA	4	25
IVB	1	6
IVC	0	0

**Table 2:** **Incidence of medical comorbidities**.

Variable	*N*
Total patients	16
**COMORBIDITIES**	
Hypertension	12
COPD/asthma	6
Coronary artery disease	4
Diabetes mellitus	4
Hypothyroidism	2
Hx of malignancy	2
HIV	1
Chronic kidney disease	1
**NUMBER CONCURRENT ILLNESSES**	
One	1
Two	5
Three	4
Four	3
Five	1
Nine	1
Ten	1
Median number of comorbidities	3

Of note, two patients received induction cytotoxic therapy prior to starting the regimen of CCRT. One patient received three cycles of Cx/docetaxel, and the other received a single cycle of paclitaxel/cisplatin/5-FU but did not tolerate it well and the induction chemotherapy was discontinued. These patients were included as the backbone of their therapy was the regimen in question (CCRT), and the addition of induction therapy would only potentially increase toxicities; therefore their inclusion would be unlikely to skew results in a positive direction. After a loading dose of Cx 400 mg/m^2^ 1 week prior to RT, on day 1 of RT, Cx was delivered at 250 mg/m^2^ along with carboplatin at an AUC of 1.5 mg/ml/min to 13 patients and at an AUC of 2 mg/ml/min to 3 patients. Only one patient, who received treatment at an AUC of 2 mg/ml/min, required a dose reduction of carboplatin. Two patients required a dose reduction of Cx, primarily due to skin toxicity. The median number of concurrent systemic therapy cycles delivered was seven (range: 3–7 weekly cycles), with one patient dying of a cardiac event 3 weeks into concurrent therapy. It was this patient who received only three cycles of chemotherapy and was treated to only 30 Gy.

Radiation therapy was given daily at 2.0–2.12 Gy prescribed to gross disease to a median total dose of 70 Gy (range: 30–74 Gy). Fifteen of sixteen patients were treated using IMRT, and 15/16 also received RT to the neck nodal regions bilaterally. The single patient who did not receive elective neck RT was treated for a newly diagnosed second primary head and neck squamous cell carcinoma nearly 20 years after being treated with RT, so field sizes were minimized accordingly.

### Toxicity

Acute toxicities were graded as per the NCI-CTCAE 4.0 and are summarized in Table 3. Overall, the regimen was well-tolerated. The maximum percent weight loss at any point during or after therapy for patients receiving this regimen had a median of 14%. Most patients who underwent PEG tube placement had their access removed at 6–12 weeks following the end of therapy. One patient died with a feeding tube in place, and another had a PEG tube in place at last follow-up. There was no incidence of PEG tube placement after the end of CCRT due to severe acute toxicities of therapy. There was one death during treatment at approximately 3 weeks due to a cardiac event; this was felt to be unrelated to treatment and the patient was excluded from the outcomes analysis but not from the toxicity analysis.

**Table 3:** **Acute toxicities**.

	Grade 0	Grade 1	Grade 2	Grade 3	Grade 4
Leukopenia	6	9	0	0	1
Anemia	9	5	1	1	0
Thrombocytopenia	15	1	0	0	0
Acneiform rash	9	1	6	0	0
Radiation dermatitis	1	1	8	6	0
Mucositis	1	0	8	7	0
Dysphagia/odynophagia	1	0	9	5	1
Xerostomia	5	3	8	0	0

### Clinical response/outcomes

**Figure 1:**
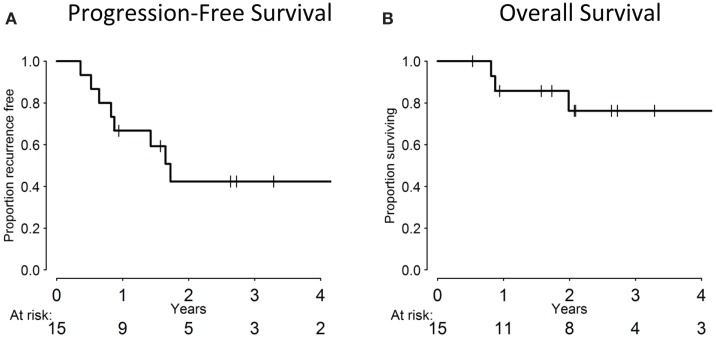
**Progression-free (A) and overall survival (B) of patient cohort**.

The median follow-up for the cohort was 24 months (range: 1–69 months). Of the 15 evaluable patients, 12 responded completely to CCRT with no residual clinical or radiographic evidence of disease, 2 responded partially, and 1 patient experienced disease progression. At the last follow-up, 8/15 patients remained disease-free. Overall, seven patients experienced a failure: three local failures, two regional failures (one of which occurred in a patient who previously experienced a local failure), and three distant failures. All local/regional failures occurred within the radiation fields. Of the distant failures, two patients metastasized to the lungs, while another developed liver metastases. All 16 patients, including the 1 who died of a cardiac event 3 weeks into treatment of non-treatment-related causes, are included in the statistical analysis, with death from unrelated cause treated as a competing risk. The 3-year cumulative incidence of LRR was 28.3% (95% CI: 7.7–53.9%) and 19.4% for distant metastases (95% CI: 4.3–42.6%). Three-year PFS was 39.7% (95% CI: 15.6–63.2%), while 3-year OS was 71.4% (95% CI: 39.8–88.4%), which are summarized in Figure 1.

## Discussion

The mainstay of therapy for patients with newly diagnosed LA-HNSCC has become RT with concurrent cisplatin-based chemotherapy. While most protocols incorporating cisplatin and RT use large bolus doses of chemotherapy (100 mg/m^2^ q3 weeks × 2–3 cycles), other regimens have used lower doses (20–40 mg/m^2^) weekly in an effort to reduce toxicities. However, neither of these regimens is feasible for a substantial number of patients with a decreased performance status or multiple medical comorbidities. While the combination of Cx/RT is often employed in this patient population and is superior to RT alone, its efficacy when compared to CRT regimens using cisplatin has been questioned (10, 11). In a recent retrospective review by Koutcher et al. from Memorial Sloan Kettering, treatment outcomes were compared between cohorts of patients receiving cetuximab/RT vs. those who received cisplatin/RT. The 2-year failure-free survival clearly favored the group receiving cisplatin (87.4 vs. 44.5%, *p* < 0.0001), as did the 2-year OS rates (92.8 vs. 66.6%, *p* = 0.0003) (10). Similar results were presented by a group from Washington University, with disease-specific survival (85 vs. 29%, *p* = 0.0012) and OS (79 vs. 20%, *p* = 0041) favoring cisplatin/RT with propensity score adjustments made for differences in baseline characteristics (11). While any retrospective analysis is subject to an inherent selection bias, these data suggest the superiority of cisplatin-based CRT regimens over Cx/RT, although this has yet to be studied in a prospective manner. Here, we present a novel treatment regimen which, to our knowledge, has not been previously reported in the literature. By adding a well-tolerated low-dose cytotoxic agent to a backbone of Cx/RT, we sought to improve treatment outcomes without adding excessive toxicity. Our results indicate that the addition of low-dose carboplatin to Cx/RT appears to produce favorable response rates while remaining a tolerable treatment regimen for this patient population.

In order to better understand the feasibility of this regimen, we compared the results in the current study to those reported in the prospective trial by Bonner et al., which established Cx/RT as a standard treatment regimen for patients with newly diagnosed HNSCC of the oropharynx, larynx, or hypopharynx. We first compared rates of Grade 3–5 toxicities, which overall revealed similar incidences between those patients treated on our study as compared to historical controls from the Bonner trial (Table 4). The lack of significantly increased toxicity with the addition of a systemic chemotherapy agent points towards the tolerability of low-dose carboplatin as well as the non-overlapping side effect profiles of the two systemic agents which were combined (9).

**Table 4:** **Comparison of toxicities**.

Toxicity (Grade 3–5)	Current study	Bonner et al. (9)
	*n* = 16	*n* = 208
Mucositis (%)	44	56
Acneiform rash (%)	0 (Highest Grade 2)	17
Radiation dermatitis (%)	38	23
Dysphagia (%)	38	26
Xerostomia (%)	0 (Highest Grade 2)	5

We also compared response rates between the current study and historical controls. Fourteen of 15 evaluable patients in our trial had either a partial or complete response, corresponding to a crude response rate of 93%. This compares favorably to the 74% CR/PR rate noted in the Bonner study. When comparing long-term control, patients in the current analysis had a 3-year locoregional control (LRC) of 71.7% (95% CI: 46.1–92.3%), vs. 47% in the Bonner trial. Three-year survival was 71.4% (95% CI: 39.8–88.4%) in our study vs. 55% in the Bonner study (9). These results are summarized in Table 5. Of note, while the distribution of primary site of disease is similar between our series and the Bonner study, our study did have a greater proportion of patients with stage III disease (63 vs. 25%), which could explain our favorable outcomes. Our results are also certainly limited by our small sample size, but they do suggest that CCRT is an effective and well-tolerated treatment approach that may warrant further prospective evaluation.

**Table 5:** **Comparison of outcomes**.

Outcome (3-year estimates)	Current study Carboplatin + cetuximab + RT *n* = 16	Bonner et al. (9) Cetuximab + RT *n* = 208
Overall response (%)	93	74
Locoregional control (%)	71.7	47
Progression-free survival (%)	39.7	42
Overall survival (%)	71.4	55

It is important to keep in perspective the patient population in whom there may be a benefit of adding low-dose carboplatin to Cx/RT. In our observational study, the regimen was only offered to patients who were not felt to be candidates for cisplatin. For those patients who are more fit, the addition of Cx to a backbone of cisplatin/RT for LAHNC was evaluated in a phase III trial by the RTOG (0522) (17). At interim analysis, there was no improvement in PFS or OS with the addition of Cx to cisplatin/RT, while there was an increase in acute toxicities. Although long-term results of this RTOG study are not yet available, it would be unlikely that these results will differ significantly given the natural history of head and neck cancers. Our study, by contrast, focused on patients who were too frail to receive full-dose of cisplatin and for whom RT plus Cx was the current standard approach. It is for this reason that we do not feel that the negative results from RTOG 0522 are relevant to this discussion. We acknowledge that combining cisplatin-based CRT with Cx may not result in an increase in therapeutic efficacy compared to cisplatin-based CRT alone. Our hypothesis is that adding carboplatin to Cx increases efficacy while not increasing toxicity significantly beyond that seen with Cx/RT.

In the studies discussed above including our cohort, the largest group of patients presented with primary tumors of the oropharynx. Over the past decade, we have become aware of changes in the biology and etiology of oropharyngeal squamous cell carcinomas, which are now more often related to exposure to human papillomavirus (HPV), as opposed to the traditional chemical carcinogens of tobacco and alcohol (18). In RTOG 0522, 73% of patients with oropharyngeal tumors had HPV-positive carcinomas, using p16 as a surrogate immunohistochemical marker for HPV positivity (17). These patients have higher rates of tumor response and long-term disease control compared to p16 negative patients, and p16 is now accepted as an independent prognostic indicator in this population (19). In the current series, all four patients with known p16+ disease remain free of disease at last follow-up. Our lack of p16 staining data for all included patients is certainly a limitation of our analysis, as it would be noteworthy to evaluate our results, or perhaps those of a planned phase II prospective trial of CCRT, in the context of HPV status.

Whether treatment can be de-intensified in HPV+ patients with oropharyngeal SCC is the focus of a phase III RTOG trial, which is currently underway (RTOG 1016), in which patients with p16+ oropharyngeal SCC are being randomized to Cx/RT vs. cisplatin/RT (18). If the results of this trial indicate non-inferiority of Cx/RT in this setting, then our CCRT regimen may not be needed for p16+ oropharynx patients. We would then focus our evaluation on the p16− population. However, if the RTOG demonstrates Cx/RT to be inferior to CRT for the p16+ population, then our regimen may still warrant evaluation in LA-HNSCC patients regardless of p16 status.

## Conclusion

For LAHNC patients who are not suitable candidates to receive cisplatin-based CRT, treatment options remain limited. By adding a low-dose of an alternative cytotoxic chemotherapy agent (carboplatin) to RT and Cx, we have identified a new treatment regimen that appears to be well-tolerated and may improve upon the therapeutic ratio of Cx/RT by increasing efficacy without adding significant toxicity. Although our results are limited by our small sample size and retrospective analysis, they are hypothesis-generating and suggest that this regimen should be evaluated in a prospective manner. A phase II trial is in development at our institution.

## Author Note

This work was presented in abstract form at the 2012 American Society of Clinical Oncology meeting, Chicago, IL, USA.

## Conflict of Interest Statement

The authors declare that the research was conducted in the absence of any commercial or financial relationships that could be construed as a potential conflict of interest.
